# A Mokken scale analysis of the Kessler-6 screening measure among Chinese older population: findings from a national survey

**DOI:** 10.1186/s12877-020-01771-w

**Published:** 2020-09-22

**Authors:** Lisong Zhang, Zhongquan Li

**Affiliations:** 1 School of Sociology and Population, Nanjing University of Posts and Telecommunications, 9 Wenyuan Road, Qixia District, Nanjing, 210023 Jiangsu China; 2 School of Social and Behavioral Sciences, Nanjing University, 163 Xianlin Avenue, Qixia District, Nanjing, 210023 Jiangsu China

**Keywords:** Psychological distress, Mokken scale analysis, Dimensionality, Differential item functioning, Sex differences

## Abstract

**Background:**

The aging population increases rapidly across the world. Timely and effective screening of their mental-health problems is important to individuals, families, and the whole society. The Kessler-6 screening measure (K6) is a very popular instrument for non-specific psychological distress. However, few studies have focused on the psychometric properties of this instrument in the older population.

**Methods:**

The present study employed Mokken scale analysis to evaluate its dimensionality and structure. This study also used differential item functioning (DIF) to examine whether the same structure existed across sex in a national representative sample of old Chinese people. Data were drawn from a public data set, the 2010 China Family Panel Studies (CFPS2010), and responses from a total of 6450 participants aged 60 years old and above (3136 males and 3314 females) were included in the final analysis.

**Results:**

Mokken scale analysis supported the unidimensional structure of the K6. Differential item functioning (DIF) analysis revealed that two of the six items (“Hopeless” and “Everything was an effort”) were marked for DIF based on the Chi-square. However, their impacts were negligible in terms of McFadden’s pseudo R^2^.

**Conclusions:**

The K6 demonstrates adequate psychometric properties in the old Chinese population. The sum of all six items can be used as an indicator of non-specific psychological distress. Differences in the indicator across sex should be considered as a real difference in psychological distress between the female and the male.

## Background

The aging population increases rapidly across the world. Mental-health problems such as depression and anxiety are prevalent in this population. They have both short-term and long-term consequences for individuals, families, and the whole society [1]. According to Report on National Mental Health Development in China (2017–2018), in the past several years, prevalence estimate of depression disorder is ranged from 15 to 39.86%, and the prevalence rate of anxiety disorder is ranged from 11.51 to 22.02% among Chinese older population [2]. Another survey with a large nationally representative sample (the China Health and Retirement Longitudinal Study (CHARLS)) also indicated that about 33.09% of Chinese older adults suffered depression disorders [3]. In consideration of the largest population and fastest aging in China [4], timely and effective screening of psychological distress is vital to help those at risk for early intervention.

The 6-item version of the Kessler Psychological Distress Scale (K6), a very brief instrument, has been developed to screen for non-specific psychological distress [5]. It was initially designed for fast and accurate detection of severe mental illness among the general population. Later, it is also used in some clinical situations [6]. It demonstrates strong psychometric properties in many populations, such as emerging adolescents [7], adults [8], and the elderly [9]. It even outperforms the K10, a long-form with ten items, in screening for DSM-IV mood or anxiety disorder [10]. Due to its excellent performance and high efficiency, it is widely employed in several major global and national surveys, such as the WHO World Mental Health (WMH) Survey, the US National Health Interview Survey [6], the Australian National Survey of Mental Health and Well-Being [10], the Canadian National Population Health Survey [11], the South African Stress and Health study [12]. It is also included in the China Family Panel Studies (Institute of Social Science Survey, Peking University, 2015), a longitudinal survey of Chinese communities, families, and individuals.

However, still some debates exist about the dimensionality of the K6, which is critical in interpreting scores on the scale. The K6 was initially developed as a measure for a unidimensional construct [5]. The one-factor solution (with all items loading on a single factor) is also supported in most of the current studies [6, 8, 11, 13–20]. Nevertheless, this model had a poor fit with the data from a large sample of adolescents in Australia, and a modified single-factor model was proposed instead [21], allowing residual correlations among some items. Moreover, two-factor models were also reported in several studies [6, 7, 22, 23]. Kessler et al. found a two-factor solution in the Indian sample, with an item (“Everything was an effort”) loading on the second factor [6]. Lee et al. examined the dimensionality of the K6 among 3014 Hong Kong residents [22]. They found a two-factor model best fit the data, with three items (“Nervous”, “Restless or fidgety”, and “Everything was an effort”) loading on the anxiety factor, another three items (“Hopeless”, “Depressed”, and “Worthless”) loading on the depression factor. Bessaha compared several models of the K6 among a large sample of emerging adults in the US and revealed that a two-factor model and a second-order two-factor model fit the data better than a one-factor model [7]. In their two-factor model, two items (“Nervous” and “Restless or fidgety”) loaded on the anxiety factor, while the other four items loaded on the depression factor. Moreover, the anxiety factor and the depression factor loaded on psychological distress in the second-order two-factor model. Easton et al. reported a better fit of Bassaha’s two-factor model than the unidimensional model to the responses from Palestinian social workers [23].

Traditionally, exploratory factor analysis (EFA) and confirmatory factor analysis (CFA) are used in examining the factor structure of the K6 [11, 21]. Mokken scale analysis (MSA) has demonstrated its unique value in addressing the problem of dimensionality [24–26]. It belongs to the family of nonparametric item response theories. It assumes that all items in a scale are hierarchically ordered along the continuum of a latent construct. It is more flexible than IRT models like the Rasch model and logistic models regarding statistical assumptions and sample size. It is not restrictive with the assumption about the sigmoid-shaped curves of item characteristics [27]. It requires a relatively small sample size to obtain a stable estimation [28]. It is also superior to traditional factorial analysis in evaluating dimensionality and models simultaneously [25]. Traditional factor analysis assumes a linear relation between items and latent construct under Classic Test Theory, and often suffers distortion from item-score distribution. In addition, factor analysis relies mainly on inter-item correlations. It assumes responses on high correlation item-pairs indicate similarities in the latent trait, which might be misleading due to some confusions, such as the similarity of wording [29].

Mokken scale analysis (MSA) evaluates the fit of two models of nonparametric item response theory to data: monotone homogeneity model (MHM) and double monotonicity model (DMM). MHM, the most general Mokken model, has assumptions of unidimensionality, local independence, and monotonicity. Unidimensionality implies that all items on the scale measure the same latent construct. Local independence implies that an individual’s response to one item is not influenced by their responses to the other items on the same scale. Monotonicity implies that an individual who has a higher trait level will always obtain a higher score on the items. DMM, a particular case of MHM, has an additional assumption of Invariant Item Ordering (IIO), which assumes nonintersection of item response functions [30]. Mokken scale analysis provides an automated item selection procedure (AISP) to help assess the latent structure of a scale [31, 32]. The total score of all items reveals different levels of the latent construct [25]. The first aim of our study is to employ Mokken scale analysis to evaluate the dimensionality of the K6.

The K6 is often used in the comparison of psychological distress across ages, sex, education, job categories, and nations [6, 11, 20, 33]. Most studies implicitly assume that the K6 measures psychological distress in the same way in different groups. However, the assumption is not always correct and should be justified before comparison [34]. Regarding the findings on the K6, women are higher than men in the mean level and prevalence of psychological distress in both adolescent and adult populations [21]. The differences may be the results of higher vulnerability and more exposure to stress events for women, or the consequences of the way they understand some items [11]. Few studies have examined measurement invariance for the K6 across sex, with the exceptions of Drapeau et al. and Mewton et al. [11, 21]. Drapeau et al. used multi-group confirmatory factor analyses testing sex invariance in different age groups with data from the Canadian National Population Health Survey. They found that though some items might vary over life-course in the sex invariance patterns, the K6 hold measurement invariance across sex in general. Mewton et al. also examined sex invariance in a sample of Australian adolescents under the framework of confirmatory factor analysis. They indicated that the data didn’t support the strong invariance model, and further examination of partial invariance models revealed that all items lacked invariance in the item thresholds. Item thresholds are related to response categories. They refer to the points along the latent trait at which transition from one response category to the next occurs, for example, from “None of the time to “A little of the time” [21]. These studies are conducted among people of different ages in western cultures. We are not sure whether the findings could be replicated in eastern cultures, such as China.

Multi-group confirmatory factor analysis is commonly used in examining measurement invariance, but it might not be accurate in figuring out the source of non-invariance. A flexible and robust iterative hybrid logit regression/ item response theory (LR/IRT) framework is recommended to deal with such a problem [35]. The logit regression approach makes comparisons among several models representing the prediction of latent trait and group membership on item performance. In addition, item response theory (IRT) models provide the estimation of latent trait scores. Simulation studies have proven the advantage of this framework in detecting DIF in comparison to other methods. Therefore, the second aim of our study is to employ differential item functioning (DIF) analysis to evaluate measurement invariance of the K6 across sex.

In all, the present study would investigate the dimensionality of the K6 and its measurement invariance across sex with data from China Family Panel Studies in the year 2010. The results would contribute to the understanding of the factor structure of the K6 in eastern cultures and shed some light on the sex difference in psychological distress.

## Methods

### Data and sample

The study is based on secondary data analysis. The data were drawn from a publicly available dataset, the 2010 China Family Panel Studies (CFPS2010). The CFPS was initiated in 2010, by the Institute of Social Science Survey of Peking University, with financial support from the Chinese government. It is an annual longitudinal survey of Chinese national representative communities, families, and individuals. It collects information related to a variety of topics, such as economic activities, education outcomes, family dynamics and relationships, migration, and health (Institute of Social Science Survey, Peking University, 2015). In the CFPS2010, a subsample of 6598 records from older adults (aged 60 and above) was selected. Due to missing responses to any K6 item, 148 records were discarded. Therefore, responses from a total of 6450 participants (3136 males and 3314 females) were included in the final analysis. Their age ranged from 60 to 110 years old, with a mean of 68.51 years (SD = 6.94).

### Measures

The K6 is among the most widely used short instruments for screening psychological distress [6]. It comprises six items related to the following feelings during the past 4 weeks, such as sad, nervous, hopeless, and worthless. Participants indicate their symptoms on a Likert scale ranged from 1 (All of the time) to 5 (None of the time). Following the instruction of the scale, we reversed the rating of the six items on a scale from 0 to 4, and summed their scores as an indicator of psychological distress. The total score ranges from 0 to 24. The higher the scores, the higher levels the psychological distress, such as anxiety and depression. The K6 has been demonstrated good reliability and validity in Chinese populations, with Cronbach’s alpha at 0.84, the 32- to 53-day interval test-retest reliability at 0.79 [15]. The Cronbach alpha coefficient is 0. 88 for the female sample, 0.86 for the male sample, and 0.87 for the whole sample in the present study.

### Statistical analyses

We performed a Mokken scale analysis to explore the factor structure of the K6 using the R package “Mokken”. The package enables us to form unidimensional subscales from all items using an automated item selection algorithm (aisp). The structure of the inventory is indicated by the pattern and scalability of each item (i.e. Hi). We also tested the assumption of local independence and the assumption of monotonicity. We were interested in measurement invariance between males and females, and we employed the “Lordif” package for R to detect differential item functioning (DIF) of items in the K6. Both uniform and non-uniform DIFs were detected with the logistic approach.

#### Examining factor structure

##### Assessment of dimensionality

We first evaluated whether the items of the K6 could form a Mokken scale in terms of scalability coefficients. The program calculated the scalability coefficient for all items in a scale (H), the scalability coefficients for each individual item in the scale (H_i_), and the scalability coefficients for each item-pair (H_ij_). In terms of Sijtsma and van der Ark [30], a scale is insufficient, if H < 0.3, weak if 0.3 ≤ H < 0.4, medium if 0.4 ≤ H < 0.5, and strong H ≥ 0.5. In addition, items in a Mokken scale also should have H_i_ values greater than 0.3 and the H_ij_ values greater than 0. We also explored the issue using the iterative automated item selection procedure (AISP). All six items were evaluated to identify potential Mokken scales. As recommend by Hemker et al. [27], we used an initial lower bound of 0, then increasing incrementally in 0.05 steps until 0.75. The c value is suggested to set at 0.3 in practice, because the solution produced by the AISP is often hard to interpret when c ≥ 0.35 [30].

##### Assessment of local independence and monotonicity

Local independence assumes that an individual’s response to one item measuring the latent construct is not influenced by their responses to the other items on the same scale. We used the conditional association procedure and calculated two indices W1 and W3 to check local independence. High W1 or W3 reveals that an item pair is likely locally dependent and violates the local independence assumption. Monotonicity implies that an individual who has a higher disposition on the latent trait will always obtain a higher score on the items measuring the latent construct. We plotted the item response function (IRF) for all six items to check monotonicity graphically. We also use the check.monotonicity() function for significance test whether the curve deviates from the monotonicity hypothesis.

##### Assessment of invariant item ordering

The Monotone Homogeneity Model and the Double Monotonicity Model mainly differ in the assumption of Invariant Item Ordering (IIO), which implies that the ordering of the items in terms of item difficulty is the same at all locations on the latent trait continuum. We attempted to check whether the Monotone Homogeneity Model or the Double Monotonicity Model had a better fit to the data. According to Sijtsma and van der Ark [30], overall IIO is insufficient if the coefficient H^T^ < 0.3, weak if 0.3 ≤ H^T^ < 0.4, medium if 0.4 ≤ H^T^ < 0.5, and strong H^T^ ≥ 0.5.

##### Reliability

In addition to Cronbach’s α and Guttman’s lambda-2 λ_2_, we also reported the Molenaar-Sijtsma (MS) reliability estimate and the Latent Class Reliability Coefficient (LCRC), which are recommended in the Mokken scale analysis. The former assumes the Double Monotonicity Model hypothesis, while the latter is robust to violation of the assumption. A scale is acceptable if all these indices are greater than 0.7 [27].

### Examining measurement invariance

Following the procedure proposed by Choi et al. [36], we conducted differential item functioning (DIF) analysis under the hybrid iterative LR/IRT framework with “Lordif” package in R. Three ordinal logistic models (models 1, 2, and 3) were established for each item involving item performance, latent trait score, group membership, and the interaction between the latter two. Model 1 is a baseline model, including only the latent trait score as the predictor. Model 2 is a uniform DIF model, including the latent trait score and group membership as predictors. Model 3 is a non-uniform DIF model, including latent trait score, group membership, and their interaction as predictors. DIF detection is based on the likelihood ratio (LR) χ^2^ test at the α level of 0.01. A significant difference in the log-likelihood values between Model 2 and Model 1 reveals uniform DIF, while a significant difference between Model 3 and Model 2 indicates non-uniform DIF. DIF magnitude is based on McFadden’s pseudo-R^2^, < 0.13 as negligible, 0.13 < R^2^ < 0.26 as moderate, > 0.26 as large [37]. Under the framework, the latent trait score was estimated by default fitting Graded Response Model (GRM).

### Additional exploratory factor analysis and confirmatory factor analysis

Moreover, we conducted exploratory factor analyses and confirmatory factor analyses to examine the factor structure of this scale using Mplus 7.4. Due to the ordinal nature of the scale, we used weighted least squares with a correction to means and variances (WLSMV) as the estimation method [29]. For the EFA, eigenvalues greater than one, scree plot, and parallel analysis were employed to determine the number of factors [38]. In addition, the goodness of model fit indices was jointly considered in both EFA and CFA. A good fit is suggested if the comparative fit index (CFI) ≥0.90, the Tucker-Lewis index (TLI) ≥0.9, and the root-mean-square error of approximation (RMSEA) ≤ 0.08 [39].

## Results

### Descriptive statistics

Responses distribution on five categories for each item in the K6 is presented in Table 1. We can see the symptoms distributed as a positive skewness. The majority of people have no symptoms, while only a few have severe symptoms. According to the cut point of 12/13, the prevalence of psychological distress among the current sample is 5.3%.

**Table 1:** Responses distribution on five categories

Item	0	1	2	3	4
1.Depressed	3521 (54.59%)	2052 (31.81%)	275 (4.26%)	431 (6.68%)	171 (2.65%)
2.Nervous	4099 (63.55%)	1782 (27.63%)	193 (2.99%)	296 (4.59%)	80 (1.24%)
3.Restless or fidgety	4007 (62.12%)	1741 (26.99%)	260 (4.03%)	328 (5.09%)	114 (1.77%)
4.Hopeless	4650 (72.09%)	1207 (18.71%)	202 (3.13%)	292 (4.53%)	99 (1.53%)
5.Everything was an effort	3813 (59.12%)	1599 (24.79%)	278 (4.31%)	545 (8.45%)	215 (3.33%)
6.Worthless	4790 (74.26%)	1142 (17.71%)	164 (2.54%)	251 (3.89%)	103 (1.60%)

Note. *N* = 6450. 0 = None of the time, 1 = A little of the time, 2 = Some of the time, 3 = Most of the time, 4 = All of the time

### Examining factor structure

#### Assessment of dimensionality

The scalability of the K6 is presented in Table 2. For inter-item pairs, the inter-item scalability coefficients (Hij) range from 0.47 to 0.68. For items, the item scalability coefficients (Hi) ranged from 0.57 to 0.59. For the whole K6 scale, the scalability coefficient was 0.58 (SE = 0.009). All the scalability coefficients were significantly greater than the conventional lower-bound value of 0.3. The results suggested the K6 should be considered as a scale of strong strength. The internal consistency of the six items was also excellent (Cronbach’s alpha =0.87).

**Table 2:** Descriptive statistics of the items (upper panel) and the scale (lower panel) for the K6

Item	M	SD	H_**j**_	SE	citc
1	0.71	1.01	0.581	0.011	0.72
2	0.52	0.86	0.555	0.011	0.70
3	0.57	0.92	0.590	0.010	0.76
4	0.45	0.88	0.584	0.011	0.74
5	0.72	1.09	0.574	0.010	0.71
6	0.41	0.85	0.592	0.011	0.74
M			3.38		
SD			4.38		
H			579	0.009	
α			0.87		
λ_2_			0.87		
MS			0.87		
LCRC			0.87		

Note. *N =* 6450*.* H_j_ = item-scalability coefficient; SE = standard error of item scalability coefficient; citc = corrected item-test correlation; H = total-scalability coefficient; α = Cronbach’s alpha; λ_2_ = Guttman’s lambda-2; MS = Molenaar–Sijtsma method; LCRC = Latent Class Reliability Coefficient

We further explored the dimensionality for all the six items by conducting iterative automated item selection procedure (AISP). The results were presented in Table 3. We followed the recommendation of Hemker et al. (1995), and set an initial value of lower bound c from 0 to 0.75 with increment steps of 0.05. For 0 ≤ c ≤ 0.55, all six items were selected to form one scale. For c = 0.6, two scales emerged, including items 1–3 and items 4–6, respectively. For c = 0.65, items 1 and 3 were unscalable. For c > 0.7, all items were unscalable. The c value is suggested to set at 0.3 in practice, because the solution produced by the AISP is often hard to interpret when c ≥ 0.35 [30]. Therefore, the results from the AISP confirmed the unidimensionality of the K6.

**Table 3:** The results of automated item selection procedure for the K6

		Item numbers		
c	Results	Scale 1	Scale 2	Unscalable
0–0.55	1: 6	1–6		
0.6	2:3,3	1–3	4–6	
0.65	2:2, 2	2, 3	4, 6	1,5
0.7–0.75	0			1–6

#### Assessment of local independence and monotonicity

**Fig. 1:**
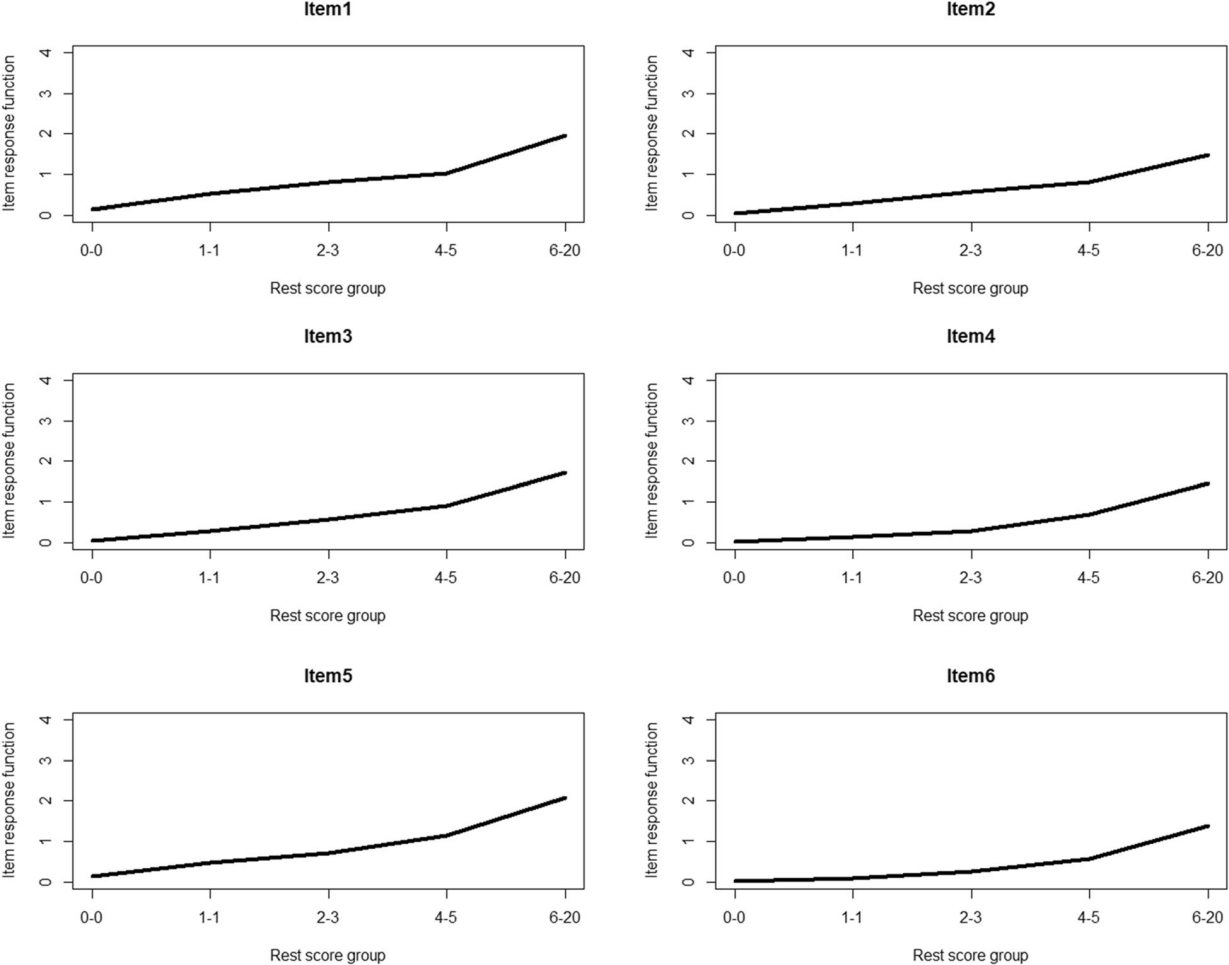
Monotonicity plots of the K6 items

Moreover, we examined local independence and monotonicity to make sure that the fit between data and the Mokken scale model was adequate. Regarding local independence, there was not any item-pair marked as locally dependent in terms of W1 and W2, two indices calculated in the conditional association procedure [30]. Regarding monotonicity, there was not any item marked as non-monotonical (see Table 4). The visual analysis suggested that all items showed monotonical increases (see Fig. 1). In brief, all items met the assumptions of local independence and monotonicity.

**Table 4:** Output of assessment of monotonicity

Item	#ac	#vi	#zsig	crit
1	40	0	0	0
2	40	0	0	0
3	40	0	0	0
4	40	0	0	0
5	37	0	0	0
6	40	0	0	0

Note. *N =* 6450*.* #ac = number of active pairs that were investigated; #vi = number of violations in which the item is involved; # zsig = number of significant z-values; crit = Crit value

#### Assessment of invariant item ordering

Graphically comparisons indicated that several IRFs were almost identical, and it was hard to establish an invariant item ordering from visual inspection. A more rigorous method, increasing in transposition [40], was employed to investigate invariant item ordering, and the results suggested that Item 5 and Item 1 showed signs of violating invariant item ordering. Coefficient H^T^ = 0.09, is much less than the conventional criteria 0.3, which means that the item ordering is too inaccurate to be useful. Therefore, the invariant item ordering assumption is not supported. In sum, the Double Monotonicity model didn’t fit the data well, while the three assumptions (unidimensionality, local independence, and monotonicity) of the Monotone Homogeneity model were still met. People can be ordered on the latent trait according to their total score on the scale.

#### Reliability

Table 2 also provides reliability-estimates: coefficients of α =0.87, λ2 = 0.87, MS = 0.87, and LCRC = 0.87. All estimates are close to .9, and thus satisfactory. The corrected item-test correlations were adequate for all items, ranged from 0.64 to 0.70.

#### Sex differences

We also conducted the same analyses for male and female subgroups separately. A similar pattern emerged for the scalability assessment among these two samples. Therefore, the K6 assesses psychological distress in a similar way and with a similar strength both sex.

#### Examining measurement invariance

**Fig. 2:**
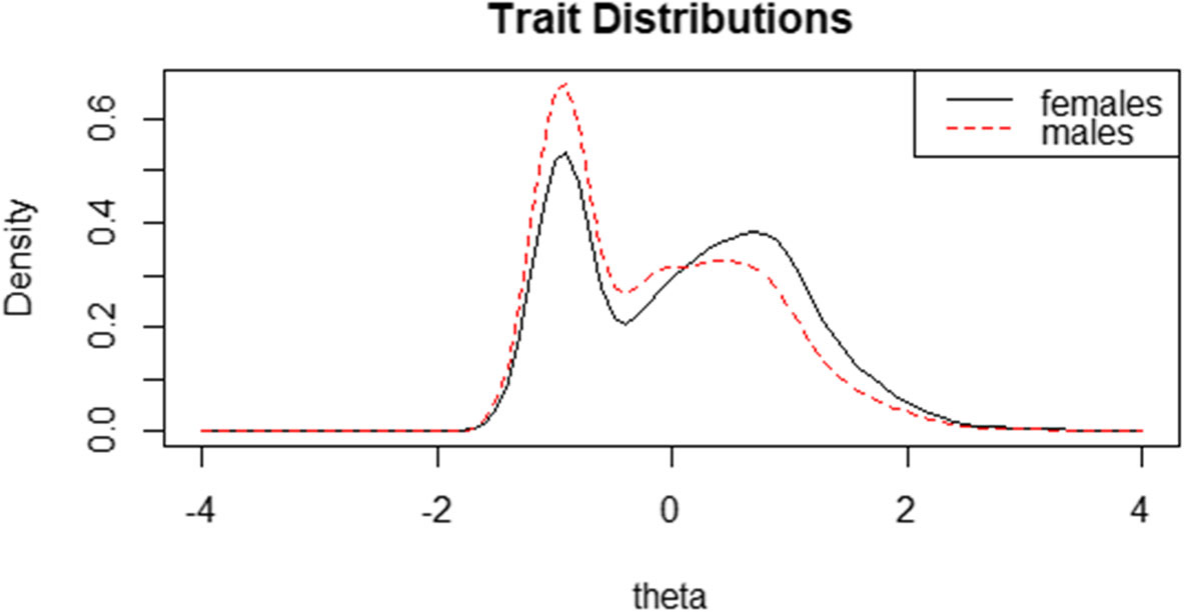
Trait distributions. Females (solid line) vs. Males (dashed line)

**Fig. 3:**
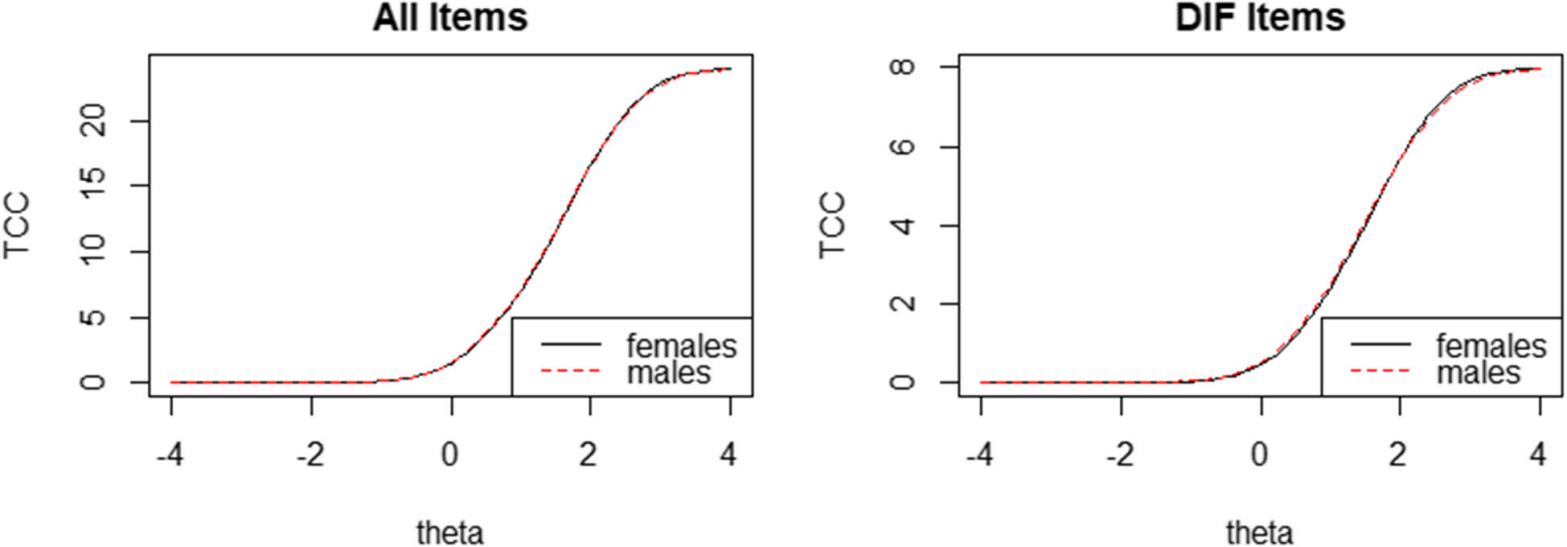
Impact of all items (left) and DIF items (right) on test characteristic curves. Females (solid line) vs. males (dashed line)

Figure 2 illustrates the trait distributions of the male and the female. The male has lower mean scores than the female, but there is still a broad overlap. Table 5 presents the main results of DIF analysis. According to the LR χ^2^ test, Item 4 and Item 5 were marked for uniform DIF, but none was flagged for non-uniform DIF. Further examination of these two items revealed that for the same latent trait score, females were always rated with higher frequencies than males. For both items, the lower-left graph shows the uniform DIF was mainly caused by the fifth category threshold value (3.31vs.2.9, 2.57 vs. 2.45). However, McFadden’s pseudo R^2^ statistics (no more than 0.0011) indicated that the magnitude of DIF was very small for each item. Figure 3 represents the impact of all items and DIF items on the whole scale. The left one shows the impact of all six items, indicating a negligible difference across sex, while the right one shows curves for the 2 DIF items, indicating that female score a bit higher when sex group-specific parameter estimates were used.

**Table 5:** Differential Item Functioning in the male and the female subgroups

Item	Uniform DIF			Non-uniform DIF	
	χ_12_^2^	ΔR^2^	Δβ12	χ_23_^2^	ΔR^2^
1	0.1523	0.0001	0.0024	0.5028	0.0000
2	0.0535	0.0003	0.0028	0.0406	0.0003
3	0.1448	0.0002	0.0018	0.0431	0.0003
4	0.0004	0.0011	0.0077	0.0240	0.0005
5	0.0056	0.0005	0.0062	0.4029	0.0000
6	0.9285	0.0000	0.0001	0.4288	0.0001

### Additional exploratory factor analysis and confirmatory factor analysis

EFA yielded only one component that had an eigenvalue greater than one (eigenvalue λ = 4.41), which explained 73.5% of the total variance. Both parallel analysis and scree plot also suggested the one-factor solution. All items had factor loadings greater than 0.8 (See Table 6). However, in terms of the goodness of model fit indices, the one-factor model didn’t fit the data well, χ2 = 1336.664, df = 9, CFI = 0.972, TLI = 0.953, and RMSEA = 0.151 (90% CI 0.144, 0.158). A two-factor model (“Depressed”, “Nervous”, and “Restless or fidgety” on the first factor, while “Hopeless”, “Everything was an effort “, and “Worthless “on the second factor) had a better and an acceptable fit to the data, χ2 = 18.912, df = 4, CFI = 1.00, TLI = 0.999, and RMSEA = 0.024 (90% CI 0.014, 0.035).

**Table 6:** Factor loadings of the K6 resulted from EFA and CFA

Item	EFA			CFA		
	One-factor model	Two-factor model		One-factor model	Two-factor model	
	Factor	Factor 1	Factor 2	Factor	Factor 1	Factor 2
1.Depressed	0.811	0.585		0.811	0.838	
2.Nervous	0.828	0.907		0.828	0.839	
3.Restless or fidgety	0.859	0.644		0.859	0.891	
4. Hopeless	0.855		0.880	0.855		0.884
5. Everything was an effort	0.802		0.671	0.802		0.839
6. Worthless	0.857		0.931	0.857		0.882

In CFA, We tested the one-factor model and the two-factor model derived from EFA, as well as three other two-factor models proposed by Kessler et al. [6], Lee et al. [22], and Bessaha [7]. In Kessler et al’s model, an item (“Everything was an effort”) loads on the second factor, while all other five items load on the first factor. In Lee et al.’s model, three items (Nervous”, “Restless or fidgety“, and “Everything was an effort“) load on the anxiety factor, while the rest three items (“Hopeless”, “Depressed”, and “Worthless“) load on the depression factor. In Bessaha’s model, two items (“Nervous“ and “Restless or fidgety”) loaded on the anxiety factor, while all the other four items on the depression factor. The model goodness-of-fit indices are displayed in Table 7. Because Kessler et al’s model is not identified in CFA, the estimation is not reliable and not listed in the table. The fit indices suggested that the two-factor model derived from EFA in the present study is the only acceptable model. Both EFA and CFA showed that the two-factor model is the best.

**Table 7:** Model goodness-of-fit indices

Model	χ2	df	CFI	TLI	RMSEA	RMSEA 90%CI
One-factor model	1336.66	9	0.972	0.953	0.151	0.144, 0.158
Two-factor model	268.736	8	0.995	0.990	0.071	0.064, 0.079
Two-factor model (Lee et al.)	1270.999	8	0.973	0.950	0.156	0.149, 0.164
Two-factor model (Bessaha)	1041.832	8	0.978,	0.959	0.142	0.134, 0.149

## Discussion

The present study employed a Mokken scale analysis on the K6 to evaluate its dimensionality and structure, and employed DIF analysis to examine whether the same structure existed across sex in a national representative sample of old Chinese people. The results confirmed the unidimensionality of the instrument and justified the sum score of all the six items as an indicator of psychological distress. Our study also supported the measurement invariance of the K6 between male and female populations.

The K6 was developed as a unidimensional measure for psychological distress at the beginning [5]. Later studies reported different factor solutions, one-factor models, and two-factor models, with exploratory factor analysis and confirmatory factor analysis in diverse samples [13, 14, 22, 41]. The incongruent findings may result from differences in populations (e.g., emerging adults and mid-age general population) and statistical methods (e.g., principal axis factor analysis, principal component analysis). Considering the J-shape distribution of item scores in the K6, we employed a new approach, Mokken scale analysis, to address the problem in older people. The approach is more flexible, relying less on item score distributions and sample size [42]. Mokken scale analysis is recommended as a more appropriate method for dimensionality assessment with discrete data [43]. In addition, previous studies mainly focused on the general population, or some specific population, such as adolescents, emerging adults, but few have taped the aging population. Our findings supported the unidimensional solution, which is consistent with the original design of the K6 and most previous studies investigating the factor structure of the K6. It contributes to the understanding of the sum score of all six items of the K6 as the indicator of psychological distress among aging populations.

Measurement invariance is the premise for group comparison [34]. Previous studies indicate that females always have more severe symptoms than males, but only a few studies have examined measurement invariance of the K6 across sex [21]. Drapeau et al. [11] and Mewton et al. [21] examined measurement invariance under the framework of confirmatory factor analysis. We explored measurement invariance under the LR/IRT framework and found two items were marked as with uniform DIF in terms of Chi-square. For Item 4 (“Hopeless”), Drapeau et al. found that women had higher first three thresholds, but lower last thresholds than men. For Item 5 (“Everything was an effort”), they only found sex invariance only in the younger age group and only at cycle 7 of the study. In the India sample, this item was separated as a second factor [6]. Mewton et al. [21] revealed that all six items had higher endorsement rates for females than males. Since the likelihood ratio test is largely influenced by sample size, DIF magnitude is also recommended to consider in detecting items with DIF. In terms of McFadden’s pseudo R^2^, the impact of the two items is negligible. Therefore, we agree with Drapeau et al. that the items in the K6 measure distress in males and females at the same degree [11]. The sex difference in the K6 scores is a reflection of the true difference in psychological distress rather than bias in reporting of the K6 items. In general, the psychological distress for females is more severe than that for males.

We also employed exploratory factor analysis and confirmatory factor analysis to explore the dimensionality of the K6. The two-factor model is the only acceptable model in comparison to other models in terms of fit indices. Our solution is somewhat distinctive from findings in other studies, partly due to the different treatment of the data and the analytical methods. Most of the previous studies treated the data as continuous, used principal axis factor analysis for exploratory factor analysis, and maximum likelihood estimator in the CFA. We conducted the analysis based on polychoric correlation with WLSMV estimator, which is more recommended due to the ordinary nature and the non-normal distribution of the data [44]. However, it might be hard to explain the solution itself: Why “Depressed” loads on the same factor with “Anxiety” and “Nervous” rather than “Hopeless” or “Worthless”? Why “Everything was an effort” loads on the same factor with “Hopeless” and “Worthless” rather than “Anxiety” or “nervous”. Factor analysis greatly depends on inter-item correlations, which may result in forming a scale in terms of insignificant factors (e.g., the similarity of wording) rather substantial relationship in the construct [29]. According to traditional indexes to determine factor numbers in EFA (eigenvalues greater than one, scree plot, and parallel analysis), the one-factor solution seemed to be more reasonable. Even in Lee et al. ‘s study, EFA results also suggested a one-factor solution: only one eigenvalue was greater than 1, and explained 56.4% of the total variance [22]. In fact, the one-factor solution is supported in most studies.

Some limitations should be acknowledged about the study. The present study among the few studies focused on examining the psychometric properties of the K6 among a relatively large and national representative sample of the Chinese older people. We only focused on the general aged population here. People in different age groups endorse the items in a somewhat different way [11, 21]. Therefore, the conclusion might not apply to other age groups. In addition, the epidemiological character of psychological distress may not be the same in different cultures [12]. We should be careful before generalization of the findings to populations in other cultures. Moreover, we only investigated the factor structure and sex invariance of the K6 here. Further studies can extend to other issues, such as screening efficiency in comparison with clinical diagnostic measurements.

## Conclusions

We employed a Mokken scale analysis on the K6 to evaluate its dimensionality and structure, and whether the same structure existed across sex in a national representative sample of older Chinese adults. The K6 demonstrates adequate psychometric properties in the old Chinese population. It measures a unidimensional construct and holds measurement invariance across sex. The sum of all six items can be used as an indicator of non-specific psychological distress and to rank people on the latent construct. Differences in the indicator across sex should be considered as a real difference in psychological distress between the female and the male.

## Data Availability

The raw data is publicly available at https://osf.io/fq8ct/?view_only=c97f6e3bc77341d2a9bbe33f67a09c60

## Abbreviations

K6: The 6-item version of the Kessler Psychological Distress Scale
DIF: Differential item functioning
LR/IRT: Logit regression/ item response theory
EFA: Exploratory factor analysis
CFA: Confirmatory factor analysis
PCA: Principal components analysis
MSA: Mokken scale analysis
MHM: Monotone homogeneity model
DMM: Double monotonicity model
AISP: Automated item selection procedure
